# Symptoms and Management of Painful Progressive Swelling in Eswatini Snakebite Patients: A Prospective Observational Study

**DOI:** 10.4269/ajtmh.24-0671

**Published:** 2025-04-15

**Authors:** Jonathan Steinhorst, Thea Litschka-Koen, Bianca Ascenção, Lindelwa Mmema, Nondumiso Shongwe, James Murray, Harry VanderWal, Rafael Cuginotti de Oliveira, Trevor Sithole, Sara Padidar, Nicholas R. Casewell, Jonathan Pons, Robert A. Harrison, David G. Lalloo, Ymkje Stienstra

**Affiliations:** ^1^ Department of Internal Medicine/Infectious Diseases, University Medical Centre Groningen, University of Groningen, Groningen, The Netherlands;; ^2^ Eswatini Snakebite Research and Intervention Centre, Simunye, Eswatini;; ^3^ Eswatini Antivenom Foundation, Simunye, Eswatini;; ^4^ The Luke Commission, Sidvokodvo, Eswatini;; ^5^ Research Unit, Ministry of Health, Mbabane, Eswatini;; ^6^ Department of Biological Sciences, University of Eswatini, Kwaluseni, Eswatini;; ^7^ Centre for Snakebite Research and Interventions, Liverpool School of Tropical Medicine, Liverpool, United Kingdom

## Abstract

In Eswatini, bites from snakes with cytotoxic venoms inflict substantial morbidity on humans through blistering, swelling, and tissue necrosis. Despite its widespread use, there is little evidence regarding the efficacy of antivenom in preventing snakebite-induced tissue damage. We conducted a prospective observational study in nine hospitals in Eswatini to describe and quantify symptoms of local tissue toxicity. Our secondary aim was to examine the use of antivenom. Data from 125 snakebite patients with extensive or rapidly progressive swelling were analyzed. The median increase in circumference of envenomed limbs was 12%. Necrosis developed in 31 (25%) patients, primarily in distal extremities. Seventy patients (56%) received South African Institute for Medical Research (SAIMR) Polyvalent antivenom (South African Vaccine producers, Johannesburg South Africa), which was administered for indications related to local tissue damage. Upon hospital presentation, patients treated with antivenom exhibited slightly more severe swelling. Ten out of 11 patients with necrosis upon admission received antivenom. At least seven patients developed necrosis after admission despite previous antivenom therapy. In this nonrandomized observational study, no relationship was observed between the rate at which swelling receded and antivenom treatment. Adverse reactions to antivenom occurred in 49% of patients. Although our analysis has its limitations, it emphasizes the compelling need for research into the indications for and outcomes of antivenom treatment for local tissue damage.

## INTRODUCTION

Globally, snakebites in humans inflict a substantial disease burden, primarily affecting low- and middle-income countries in the global South.^1^ One-third of global venomous snakebites are thought to occur in sub-Saharan Africa, where annual cases of snakebite envenoming range from 91,000 to 420,000, with mortality remaining largely unknown; however, estimates range from 3,500 to 32,000 deaths.^2^^,^^3^ Underreporting of cases and the non-recognition of snakebite as a disease entity indicate that the true incidence is likely higher.^2^^,^^4^^,^^5^ Bordered by South Africa and Mozambique, Eswatini is a landlocked country with 1.2 million inhabitants.^6^ Clinical studies on snakebite from Eswatini are unavailable, but recently published data^7^ from public health registries show an average of 466 snakebites reported annually at health facilities across the country. Moreover, reports by the Eswatini Antivenom Foundation^8^ and qualitative research^9^^,^^10^ underscore that snakebites are a pressing issue.^11^^,^^12^

Local tissue damage is a major driver of snakebite-related morbidity and disability.^13^^,^^14^ Although snake identification remains a challenge, syndromic assessment of bitten patients, combined with the distribution of snakes identified in the region, suggests that cytotoxic snakebites in Eswatini can be ascribed to *Naja mossambica* (*N. mossambica*; Mozambique spitting cobra; family Elapidae), *Bitis arietans* (*B. arietans*; puff adder; family Viperidae), *Hemachatus hemachatus* (*H. hemachatus*; Rinkhals; family Elapidae), *Atractaspis bibronii* (*A. bibronii*; stiletto snake; family Atractaspididae), *Causus rhombeatus* (*C. rhombeatus*; rhombic night adder; family Viperidae), and *Causus defilippii* (*C. defilippii*; snouted night adder; family Viperidae).^8^^,^^15^^,^^16^ The hallmark signs of *N. mossambica* envenoming are swelling, pain, superficial tissue necrosis, and blistering at the bite site.^17^^,^^18^ Compared with *N. mossambica, B. arietans* has longer fangs, resulting in deeper tissue penetration, and its venom contains hematotoxins in addition to cytotoxins.^19^ Envenoming by this snake manifests as swelling, blistering, necrosis, and ecchymosis surrounding the bite site, as well as coagulopathy and bleeding in some reports.^19^^–^^24^ *Hemachatus hemachatus* venom is both cytotoxic and neurotoxic, sometimes causing superficial necrosis and progressive weakness.^18^ Envenoming by *A. bibronii*, *C. rhombeatus,* and *C. defilippii* is not associated with systemic effects but rather with mild to moderate local symptoms. Existing antivenoms do not include their venoms in production.^25^ In contrast, envenoming by *N. mossambica*, *B. arietans,* and *H. hemachatus* is generally more severe, resulting in significant tissue damage and morbidity,^15^^,^^19^^,^^26^^,^^27^ and their venoms are used in antivenom production.^25^^,^^28^

A number of studies and reports have suggested that antivenom treatment is associated with less necrosis,^17^^,^^19^^,^^29^ less pain,^30^ a reduction in intra-compartmental pressure,^31^^–^^33^ and a lower incidence of fasciotomy.^34^ However, the effects of antivenom on local envenoming have rarely been formally evaluated in randomized clinical trials. Snakebite management guidelines by the WHO^27^^,^^35^ and the Eswatini Ministry of Health^18^ both cite severe swelling, particularly in digits, and signs of tissue damage (e.g., blistering, necrosis) as indications for antivenom therapy but do not recommend it in cases where swelling is mild (i.e., involving less than half of the bitten limb, with no rapid progression of swelling). Abouyannis et al.^36^ reported that only one-third of snakebite trials included outcome measures related to local tissue damage, with only a single study recording severe outcomes of local tissue destruction,^37^ such as debridement, skin grafting, and amputation. Clinical data on the progression of local tissue swelling and necrosis, as well as the degree to which antivenom therapy can influence its course, are urgently needed. Here, we aimed to define the clinical course of local envenoming after snakebite envenoming in patients from Eswatini, with the secondary intention of investigating whether antivenom modified the clinical course in this cohort.

## MATERIALS AND METHODS

### Data collection.

An 18-month prospective observational study was conducted over two snakebite seasons (October–March) from October 2020 through March 2022 at nine health facilities in Eswatini. These included four government-operated health centers (Dvokolowako Health Centre, Emkuzweni Health Centre, Matsanjeni Health Centre, Sithobela Rural Health Centre), four government-operated hospitals (Good Shepherd Hospital, Mankayane Government Hospital, Piggs Peak Government Hospital, Raleigh Fitkin Memorial Hospital), one of which is jointly run with a mission (Good Shepherd Hospital), and one hospital (The Luke Commission) run by a nongovernmental organization. Each of the nine health facilities was staffed with one research assistant from the Eswatini Antivenom Foundation to recruit patients and oversee data collection for the study.

All patients presenting within 24 hours after the bite with discoloration indicating necrosis, extensive swelling (more than half the bitten limb), or rapidly progressive swelling were included. Rapidly progressive swelling was defined as swelling that extends more than 5 cm per hour, swelling of the whole hand or foot within 1 hour after envenoming, swelling that extends to the elbow or knee within 3–4 hours after envenoming, or swelling of the whole limb within 8 hours after envenoming. Whether patients met these criteria was determined centrally to ensure uniform application of enrollment criteria across the nine study sites. Patients were excluded if any of the following criteria applied: 1) presentation >24 hours after the snakebite, 2) only systemic symptoms present at hospital presentation, or 3) the implicated snake was identified as a mildly or nonvenomous species.

The following patient information was collected: 1) demographics, 2) circumstances of the snakebite, 3) first aid measures, 4) available medical history, 5) local and systemic symptoms upon presentation, 6) treatment administered, 7) outcomes/complications, and 8) adverse reactions to antivenom, classified as mild (facial edema, pruritus, urticaria, fever if temperature was >38°C, rigor), moderate (abdominal pain, nausea, vomiting, bronchospasm, stridor), or severe (drowsiness/altered consciousness, confusion, hypotension with systolic blood pressure [BP] <80 mm Hg, desaturation with arterial oxygen saturation [SaO_2_] ≤92%). If available, snake identification was based on photographs of the snake or inspection of the dead snake brought to the hospital.

Clinical assessment included signs of systemic envenoming, such as hemorrhage and neurotoxicity, as well as local manifestations, which were reported in terms of swelling, loss of tissue (ulceration and necrosis), and the presence and quantity of blisters. The dimensions of bullae were measured using the maximum width and length of individual lesions. If multiple bullae were present, the largest bulla was selected for measurement. Swelling was defined as the relative increase in limb circumference of the bitten limb compared with the non-bitten limb at the point of maximal swelling. The patient’s own non-affected contralateral limb thus functioned as a control. The difference in limb circumference measurements (in cm) was expressed as a ratio (limb circumference measurement ratio [LCMR]), with the measurement of the bitten limb in the numerator and that of the non-bitten limb in the denominator. A more positive ratio of limb circumference measurements is therefore synonymous with more profound swelling. The proximal and distal extent of swelling was assessed by measuring the distance between the maximal point of swelling (i.e., the maximal limb circumference) and the anatomic height along the limb at which the limb circumference of the affected limb equals that of the non-affected limb. When feasible, photographs of the bite site were taken upon presentation and subsequently during scheduled clinical assessments. The clinical assessment was first performed upon presentation to the hospital and repeated twice at 12-hour intervals up to 24 hours, after which assessments were repeated at intervals of 24 hours. When antivenom was used, the type, dosage, dosing frequency, and primary indication for its use were recorded. Data collection at the clinical level was collated in electronic case report forms that were encrypted and uploaded to a password-protected KoboToolbox server (Kobo, Cambridge, MA).^38^ The Strengthening the Reporting of Observational Studies in Epidemiology guideline for reporting cohort studies was followed (Supplemental Table 1).

## STATISTICAL ANALYSES

We anticipated the inclusion of between 110 and 140 snakebite patients during the study period. The demographics of snakebite patients and general characteristics related to the snakebite were analyzed using descriptive statistics. Statistical comparisons of demographic characteristics and sample parameters between the antivenom group (patients who received antivenom) and the no-antivenom group (patients who did not receive antivenom) were performed using both parametric and nonparametric tests.

A Kaplan–Meier analysis was performed to compare the reduction in limb swelling between the antivenom group and the no-antivenom group over time. All patients with an LCMR ≥1.05 were classified as having swelling. The cutoff of 1.05 was chosen to account for measurement error based on an article by Bishop and Pitchey,^39^ who observed total measurement errors for mid-upper arm circumference to be 3.92–4.92% (intra- and interobserver variability) for male and female subjects sampled from the general population, respectively. Patients were included in the Kaplan–Meier analysis if their LCMR was ≥1.05 during the first 12 hours after hospital presentation. It was assumed that swelling arising with a delay of >12 hours was less likely to be venom-related and had a higher likelihood of being caused by other factors (e.g., infection), thus being less amenable to antivenom therapy. Patients undergoing operative procedures on affected limbs within the first 5 days of hospital admission were excluded from the Kaplan–Meier analysis because of concerns that surgical intervention could affect swelling. Individual missing serial observations (up to a maximum of two consecutive measurements, once per patient) were imputed using backward filling for <5% of all observations, ensuring a conservative estimate of the time until swelling diminished.

## RESULTS

During the study period, 652 patients with snakebites presented to the participating hospitals, and 173 met the criteria for painful progressive swelling. Research assistants were informed about 125 patients (72%), all of whom consented to participate in this study. Ninety-five patients (*n* = 95; 76%) were enrolled in the first snakebite season (October 2020–April 2021), whereas the remainder (*n* = 30, 24%) were included in the second season (September 2021–March 2022). Demographics of the patients and details of the bite incidents are presented in Tables 1 and 2. Comorbidities were self-reported in two patients with type 2 diabetes mellitus and 17 patients who were HIV-positive. Reliable information on the snake species involved in the bites was only available for 13 (10%) patients, of whom six were bitten by *B. arietans*, four were bitten by *N. mossambica*, and three were bitten by *A. bibronii*. A summary of health-seeking behavior and first aid methods applied is presented in the Supplemental Table 2. The median time that elapsed between the bite and arrival at the hospital was 3.3 hours (interquartile range [IQR] 1.75–5.92 hours).

**Table 1:** Demographic characteristics of snakebite patients

Demographic Characteristics of Snakebite Patients		Patients, *N* = 125 (100%)
Sex	Male	73 (58.4)
	Female	52 (41.6)
Occupation	Child/student	68 (54.4)
	Farmer	7 (5.6)
	Other formal employment	15 (12.0)
	Unspecified	35 (28.0)
Activity during the bite	Walking	47 (37.6)
	Sleeping	26 (20.8)
	Working/domestic activities/residing in and around house*	19 (15.2)
	Farming	13 (10.4)
	Playing	8 (6.4)
	Other^†^	12 (9.6)

* Includes activities such as toilet visits, cleaning, and collecting water.

^†^ Other activities included sweeping, working, sitting in an outside setting, swimming, and fixing a car.

**Table 2:** Comparison of patients treated with and without antivenom at the time of hospital presentation

Characteristics	No Antivenom, *n* = 55 (44%)	Antivenom, *n* = 70 (56%)	Total, *N* = 125 (100%)	*P*-Value
Age, median (IQR)	21 (14–29)	19 (10–34)	19.0 (12.0–32.0)	0.621*
Sex				
Male	32 (25.6)	41 (32.8)	73 (58.4)	1.000^†^
Presenting in				
First snakebite season	47 (37.6)	48 (38.4)	95 (76.0)	0.035^†^
Second snakebite season	8 (6.4)	22 (17.6)	30 (24.0)	–
Time in hours between bite and arrival at hospital, median (IQR)	2.83 (1.5–4.5)	3.50 (2.0–6.3)	3.28 (1.75–5.92)	0.204*
Patients with visible fang marks^‡^				
Yes	39 (31.2)	47 (37.6)	86 (68.8)	0.700^†^
No	16 (12.8)	23 (18.4)	39 (31.2)	–
Distance between puncture marks in mm, median (IQR)^§^	9 (3–10)	10 (5–14)	10 (5–11)	0.094*
Maximal extent of swelling in mm, median (IQR)^‖^	165 (80–240)	195 (130–320)	180 (100–310)	0.041*
LCMR, median (IQR)^¶^	1.11 (1.05–1.16)	1.14 (1.07–1.21)	1.12 (1.06–1.20)	0.061*
Necrosis				
Yes	1 (0.8)	10 (8.0)	11 (8.8)	–
No	54 (43.2)	60 (48.0)	114 (91.2)	0.022^#^
Blistering				
Yes	3 (2.4)	4 (3.2)	7 (5.6)	1.000^#^
No	52 (41.6)	66 (52.8)	118 (94.4)	–
Surgical intervention	–	11 (8.8%)	11 (8.8%)	0.004^#^
Debridement	–	9 (7.2%)	9 (7.2%)	0.004^#^
Blister deroofing	–	2 (1.6%)	2 (1.6%)	0.499^#^
Skin grafting	–	5 (4.0%)	5 (4.0%)	0.061^#^
Escharotomy**	–	2 (1.6%)	2 (1.6%)	0.500^#^
Amputation	–	1 (0.8%)	1 (0.8%)	1.000^#^
Deaths	2 (1.6%)	1 (0.8%)	3 (2.4%)	0.590^#^

IQR = interquartile range; LCMR = limb circumference measurement ratio.

* Mann–Whitney *U* test.

^†^ Pearson χ^2^ test.

^‡^ For patients with two bites, puncture marks were only counted once.

^§^ In cases with two bites, the larger measured distance was used.

^‖^ Measured as the distance in the proximal or distal direction on the affected limb in mm from the maximal point of swelling to the point of equal limb circumference (compared with the non-bitten limb).

^¶^ Measured as the circumference of the bitten limb at the point of maximal swelling and compared with the circumference of the non-bitten limb at an equivalent height.

^#^ Fisher’s exact test.

** A surgical procedure in which the constrictive effect of necrotic or burnt tissue is released.

### Systemic symptoms.

One patient demonstrated symptoms of severe neurotoxic envenoming, including fasciculations, respiratory paralysis, and limb weakness. Overall, 22 (18%) patients experienced hemorrhage: 21 from the bite site or old wounds, and one patient who suffered from gastrointestinal blood loss.

### Local symptoms upon hospital presentation.

**Figure 1.**
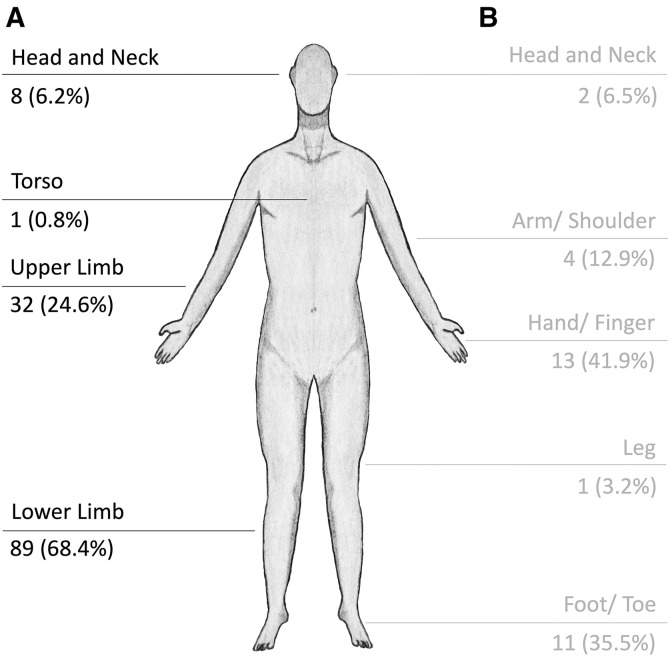
(**A**) Anatomic locations of 130 snakebites (125 patients with 130 bites; five patients had two bites). (**B**) Anatomic locations of necrosis that developed within the first 5 days of hospital presentation in a total of 31 snakebite patients.

The anatomical sites of the 125 patients affected by 130 snakebites are illustrated in Figure 1. Five patients (4%) sustained two separate snakebites on the same limb during their snake encounter. Puncture marks were visible in 90 of 130 bites (69%). Grossly visible swelling at the bite site was noted in 121 patients (97%) at the time of hospital presentation, and the median LCMR was 1.12 (IQR 1.06–1.20), signifying a 12% increase in limb circumference in the bitten extremities.

Upon hospital presentation, 11 patients (9%) were reported to have necrotic or sloughy tissue at the bite site. Of these patients, nine arrived within 4 hours of the snakebite, and nine had sustained bites in the distal extremities (foot, toes, or fingers). Bite site bullae were noted in seven (6%) patients at the time of hospital presentation, two of whom had bullae that contained blood. At hospital presentation, bullae diameters ranged from 10 to 50 mm (min–max), and the maximum distance recorded between a bulla and the bite site was 180 mm.

### Progression of local symptoms after hospital presentation.

**Figure 2.**
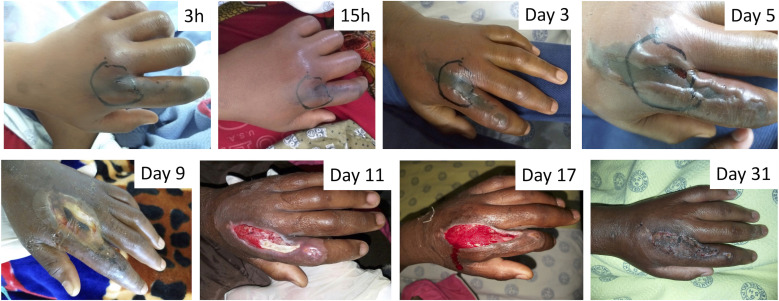
Images showing the progression of tissue necrosis in a patient bitten by a light brown snake of ∼50 cm in length (species unknown) on the left hand. The bite was sustained on the dorsal aspect of the second ray, approximately at the height of the metacarpophalangeal joint. A tourniquet was applied to the affected limb and the bite site before the patient attended the hospital. The patient presented to the hospital 3 hours after the bite and received a single dose of five vials of SAIMR Polyvalent antivenom 3 hours and 45 minutes after the bite. Necrotic skin was debrided on day 10 (after the bite), and a skin graft was performed on day 24. Images are labeled according to the time elapsed after the bite. Images are published with permission from the patient and legal guardian.

At 12 hours and 72 hours after hospital presentation, the number of patients with necrosis increased to 23 (18%) and 31 (25%), respectively (Figure 2). No patient developed necrosis on days 4 and 5 of hospitalization. In 77% of cases (*n* = 24), tissue necrosis was distal to the wrist or ankle joint (Figure 1). Twelve hours after hospital presentation, 19 additional patients had developed bullae, and 1 day after hospital presentation, bullae had formed in an additional six patients. Altogether, a total of 30 patients (24%) developed bite site bullae (eight filled with blood) within the first 4 days.

### Treatment.

#### Antivenom treatment.

Seventy patients (56%) received treatment with South African Institute for Medical Research (SAIMR) polyvalent antivenom (South African Vaccine producers, Johannesburg South Africa), which was the only product registered in the country at the time of the study. Fifty-five patients (44%) did not receive antivenom because the team concluded it was not indicated in cases in which the bite was caused by a Mozambique spitting cobra more than 12 hours before hospital admission (following the standard practice of the clinical team) or when the suspected snake was a stiletto snake or night adder. Some patients did not receive antivenom because of stockouts, a common occurrence in some facilities in Eswatini.^18^ Reasons for not providing antivenom to individual patients were not collected. The demographic and clinical characteristics of patients who did or did not receive antivenom are presented in Table 2. The anatomic locations of snakebites were comparable between the antivenom and no-antivenom groups, except that seven of the eight patients with bites on the head and neck region received antivenom, as did the only patient with a bite on the torso (Table 2). Upon admission, patients in the antivenom group, on average, had more pronounced swelling than those in the no-antivenom group. Ten of the 11 patients (91%) who presented with necrosis received antivenom. Of the remaining 20 patients who developed signs of necrosis only after admission, 18 received antivenom during the course of treatment. In at least seven of these cases, antivenom was administered before signs of visible necrosis developed. Overall, antivenom was given to 28 of the 31 (90%) patients who either had or developed necrosis after the snakebite. The median delay between the bite and the administration of antivenom was 8.8 hours (IQR 3.8–17.3 hours) for all patients who received antivenom (*n* = 70), 8 hours (IQR 5.8–15.2 hours) for the seven patients who received antivenom before visible necrosis developed, and 6.4 hours (IQR 3.2–15.2 hours) for the 24 patients who received antivenom after necrosis developed. Indications for antivenom administration per dose given are shown in Table 3. Multiple indications for administering antivenom could be selected by clinicians.

**Table 3:** Antivenom treatment

Indications for Antivenom Treatment*	First Antivenom Dose	Second Antivenom Dose	Third Antivenom Dose
No. of patients	70 (100%)	10 (100%)	1 (100%)
Rapidly progressive swelling	57 (81.4%)	9 (90%)	1 (100%)
Swelling in fingers/toes	23 (32.9%)	2 (20%)	–
Swelling > half of a limb	17 (24.3%)	2 (20%)	–
Swelling across two joints	4 (5.7%)	1 (10%)	–
Hypotension	2 (2.9%)	1 (10%)	–
Bleeding/coagulopathy	4 (5.7%)	–	–
Neurotoxicity	1 (1.4%)	–	–

* Several indications are possible per dose given.

Most patients (56%) who received antivenom were given an initial dose of five vials of SAIMR Polyvalent, the starting dose specified by the Eswatini National Snakebite Management Guidelines.^18^ In contrast, 41% of patients in the antivenom group received fewer than five vials. Of the 28 patients who developed necrosis within 5 days of hospital presentation and received antivenom, 21 (75%) were given five vials of antivenom initially, whereas seven received fewer than five vials. Of the seven patients who received antivenom before manifesting signs of necrosis, all except one received at least five vials. The largest initial dose of antivenom administered was 10 vials for rapidly progressive swelling. The highest overall amount of antivenom given to a patient was 14 vials, consisting of a first dose of 10 vials and a second dose of four vials, both for rapidly progressive swelling.

**Figure 3.**
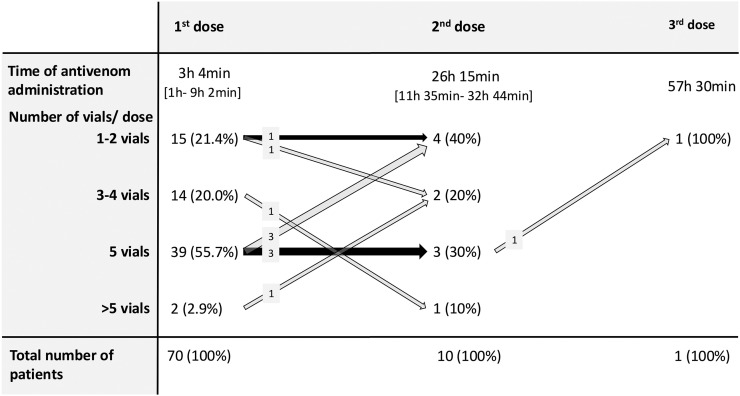
Flow diagram representing the quantity of repeated antivenom dosing.

The quantity and timing of the antivenom administered in the second dose are shown in Figure 3. Three patients received second doses of two vials of antivenom in accordance with the recommendations of the Eswatini National Snakebite Management Guidelines, and one patient was given a single vial. Repeat doses of antivenom were all administered for progressive swelling. Overall, antivenom therapy was escalated during the second round for only two patients; one patient received three doses of antivenom, totaling 12 vials altogether.

A Kaplan–Meier analysis was performed, which included 101 patients, of whom 60 had received antivenom. Twenty-four cases were excluded from the Kaplan–Meier analysis for the following reasons: absent serial observations (*n* = 13), swelling never reaching an LCMR ≥1.05 (*n* = 7), and swelling with LCMR ≥1.05 only after more than 12 hours after hospital presentation (*n* = 4). Missing serial observations were imputed 15 times (out of 460 total observations). No statistically significant difference in the rate of swelling reduction between patients in the antivenom and no-antivenom groups was found (log-rank test, *P* = 0.837). A post hoc subgroup analysis was performed on 28 patients (19 with antivenom; nine without antivenom) in the upper quartile of LCMRs (≥1.20) to further study the rate at which tissue swelling receded in the most severely envenomed category of patients. Of the 19 patients who received antivenom, 15 (79%) received a total dose of five vials or more. The Kaplan–Meier survival curves did not substantially differ from one another in this subpopulation and crossed each other multiple times during the study period.

### Other interventions.

Eleven patients, all of whom were in the antivenom group, underwent some form of surgical treatment (Table 2). The difference in the number of procedures performed between the antivenom group and the no-antivenom group was statistically significant (*P* = 0.004; Fisher’s exact test). No fasciotomies were performed during the study period.

### Outcomes and sequelae.

Three patients, all children, died as a consequence of the snakebites they incurred. The first patient presented with bruising and swelling after sustaining a bite on the foot while herding. The snake could not be identified, the patient did not receive antivenom, and the exact cause of death remained unknown. The second patient was bitten on the neck by a puff adder while asleep. This patient did not receive antivenom and died of an unknown cause while being transferred to another facility. The third patient sustained a bite (snake species not identified) on the head and neck region while asleep, after which the patient developed rapidly progressive swelling. Five vials of antivenom were administered, but the patient later died, with the exact cause of death not recorded.

Adverse reactions to antivenom occurred in 34 patients (49% of all patients receiving antivenom), with a median age of 14 years (IQR 9–33 years). Mild reactions were the most frequent (*n* = 32), predominantly urticaria (*n* = 22) and pruritus (*n* = 16). Seven patients experienced moderate reactions, and seven patients experienced severe reactions to antivenom. Severe reactions included hypotension (systolic BP <80 mm Hg) in five patients, desaturation (SaO_2_ ≤92%) in three patients, and confusion in three patients.

## DISCUSSION

The clinical presentation, management, and outcomes of local tissue destruction have long been poorly understood and under-researched, despite many venomous snakes inflicting bites on humans and possessing myriad cytotoxic venom components implicated in necrosis and swelling.^16^^,^^17^^,^^40^^–^^42^ This is particularly true for Eswatini, where snakebites in humans are primarily caused by snakes with cytotoxic venom, such as *N. mossambica*, *B. arietans*, and, less frequently, *H. hemachatus* and *A. bibronii*.^18^^,^^41^ Nevertheless, prospective clinical data on the progression of local tissue damage after cytotoxic snakebites are extremely limited globally, and no such studies are available from Eswatini to date. Furthermore, efficacy endpoints for antivenom in nonhuman preclinical studies and the few existing snakebite trials are most commonly related to survival,^43^^,^^44^ whereas outcome measures for tissue damage are less widely used and employ heterogeneous pathology criteria.^36^^,^^45^ Understanding the relative frequency and quantifying the extent of local tissue complications is an essential prerequisite for defining endpoints related to tissue damage in future clinical snakebite studies.

In this study, we present prospective observational clinical data on the physical appearance and progression of local tissue envenoming in 125 hospitalized snakebite patients in Eswatini, all of whom experienced painful progressive swelling.

The large majority (93%) of bites were sustained in the extremities, with two-thirds of all bite sites located in the lower leg. The anatomical distribution of snakebites conforms with available observational data from Eswatini^7^ and South Africa.^15^^,^^16^^,^^46^ In this group of patients with painful progressive swelling, the median enlargement in bitten limb circumference was 12% compared with contralateral healthy limbs at the time of hospital presentation. This is substantially lower than the values reported by Wood et al.,^42^ who, by means of sonography, determined expansion coefficients (the ratio of tissue compartment diameter in the bitten limb versus the healthy limb) for subcutaneous tissues and muscle compartments of 2.0 and 1.06, respectively, in 42 snakebite patients from KwaZulu-Natal, South Africa. The difference may, in part, be explained by different measurement techniques; swelling in individual tissue compartments is likely asymmetrically skewed toward the bite site and does not translate into full 360-degree radial enlargement. The corresponding change in limb circumference may therefore be less pronounced, resulting in a more modest ratio when compared with healthy limbs. In addition, the authors used a convenience sample, thus likely selecting a different patient category than in our study. The use of ultrasound in diagnosing local swelling and tissue damage is still in its infancy but has been shown to be beneficial in discriminating between swollen and non-swollen tissue compartments and, more recently, in estimating the progression of local tissue swelling over time.^47^ Nevertheless, ultrasound requires training and is unavailable to many lower-level health centers.

The time of onset of necrosis was variable, with the number of patients exhibiting signs of bite site necrosis nearly tripling between the first observation and 72 hours post-admission. The cause of this variation is unclear but could be related to differences in snake species and their respective venom composition, the quantities of venom injected, or modifications due to antivenom therapy. We were unable to reliably identify the snake species in most patients; however, such high rates of necrosis are consistent with *N. mossambica* and *B. arietans* bites.^8^ Necrotic lesions were most likely to occur in the digits, hands, and feet. This was particularly true in the subgroup of patients who already had established necrosis at the time of hospital presentation. Limited soft tissue coverage, a higher ratio of venom to tissue, limited lymphatic and venous drainage of venom,^16^ and perfusion being more susceptible to failure^40^^,^^48^ could explain this finding.

More than half (56%) of all patients in the study sample were treated with SAIMR Polyvalent, the only available antivenom in Eswatini.^41^ Using a Kaplan–Meier analysis, we were unable to find a difference in the rate of swelling reduction over time between patients receiving antivenom and those who did not. Overall, antivenom was used in the majority (90%) of patients who either had necrosis on admission or developed it thereafter, and in at least seven cases, it was administered before visible signs of necrosis developed. This finding raises a number of questions that warrant further discussion.

SAIMR Polyvalent is manufactured using venom from the 10 (*B. arietans, Bitis gabonica, H. hemachatus, Dendroaspis angusticeps, Dendroaspis jamesoni, Dendroaspis polylepsis, Naja nivea, Naja melanoleuca, Naja annulifera, and N. mossambica*) most clinically relevant and commonly encountered cytotoxic and neurotoxic snakes native to Eswatini and South Africa (package insert, Jan. 2006). Several regionally endemic snakes (*A. bibronii*, *C. rhombeatus*, *C. defilippi*), which are known to cause symptoms of mild to moderate cytotoxic envenoming near the bite site, are not included in the manufacture of SAIMR Polyvalent. Any attempt to treat local tissue damage in such patients with antivenom would likely have proven less effective, or even entirely ineffective. The high proportion (90%) of unidentified snake species in this study sample underscores the need to develop molecular and immunodiagnostic tools for snake species identification in sub-Saharan Africa. Reliable identification of the biting species would be instrumental in establishing the validity of future clinical research into the effectiveness of species-specific treatments, such as antivenoms.^49^

Using snake venom from local and regional snakes in production is essential to maximize the efficacy of antivenom because snake venom can exhibit significant geographic and taxonomic variation.^50^^–^^52^ In vitro tests of SAIMR Polyvalent have shown that it broadly binds to diverse venom proteins^41^^,^^50^ and is capable of neutralizing the activity of important cytotoxic enzymes, including those from wild-caught Eswatini snakes.^41^ Menzies et al.^41^ also demonstrated that SAIMR Polyvalent can prevent dermonecrosis in mice. Interestingly, the highest dose of antivenom was required to prevent the cytotoxic effects of *N. mossambica* venom, a snake likely to be implicated in a large proportion of cytotoxic snakebites in Eswatini. Such preclinical efficacy studies are essential to identify promising antivenom candidates and are recommended by the WHO.^43^ However, such models cannot be directly extrapolated to human cases of snakebite envenoming.^52^ The preincubation of venom–antivenom mixtures^41^ artificially optimizes conditions for venom–antivenom interaction while eliminating treatment delay. The delayed administration of antivenom (median 9 hours) in our patients could have contributed to the absence of any noticeable reductions in swelling after antivenom was given. The murine dermonecrosis model^41^ is also pharmacokinetically distinct from a clinical treatment setting because antivenom (together with venom) is injected intradermally^41^^,^^43^ and not intravenously, as is done in clinical practice. The wide-ranging tissue necrosis and microvascular thrombosis caused by cytotoxic venoms lead to a loss of functional tissue architecture,^40^^,^^53^ impede perfusion, and likely hamper the infiltration of antivenom from the circulation into the bite site. In support of this theory, Yang et al.^53^ observed diminished necrosis after subcutaneous and intramuscular antivenom injection (compared with the intravenous route) in mice previously exposed to *Naja atra* venom. Similarly, in a murine study investigating the inhibition of dermonecrotic effects of *Naja nigricollis* envenoming using the phospholipase A2-inhibitor varespladib, Bartlett et al.^54^ found that intradermal administration of varespladib significantly reduced the size of necrotic skin lesions, whereas intravenous administration did not. Importantly, lesion size was smallest when varespladib was administered immediately after venom challenge, emphasizing that the early neutralization of venom toxins is crucial in limiting dermonecrosis.^17^^,^^55^

In this study, clinicians adhered to the starting dose recommended by the Eswatini National Snakebite Management Guidelines for bites by *N. mossambica, H. hemachatus, and B. arietans* in 56% of cases. However, in >40% of cases, fewer than five vials were administered during the first round. This may be related to a combination of factors. Firstly, treating physicians may have given less antivenom for milder cases, particularly given that SAIMR Polyvalent is a costly product with a limited supply.^41^^,^^50^ This is consistent with the finding that only 10 of the initial 70 patients receiving antivenom were given additional doses. Secondly, SAIMR Polyvalent has frequently been associated with adverse reactions of varying severity,^15^^,^^16^^,^^56^^–^^59^ which our results corroborate. Clinicians may assume a dose-dependent relationship regarding such reactions,^59^ prompting them to administer smaller quantities initially and escalate therapy as needed and once deemed safe or potentially halt antivenom infusion after the occurrence of adverse reactions. The high rate of observed adverse reactions to SAIMR Polyvalent and other antivenoms in general^60^^,^^61^ emphasizes 1) the need to define clear indications for when antivenom is beneficial, 2) the necessity of investigating the etiology and pathophysiological nature of these reactions, and 3) the urgent need to develop safer antivenoms or novel therapies that are safe to use, particularly in an out-of-hospital setting.

### Strengths and limitations.

The data gathered from 125 patients yield important insights concerning the demographics and clinical features of patients with painful progressive swelling after a snakebite. Several limitations of this study should be mentioned. It was impossible to reliably identify the vast majority of biting snakes—a challenge most studies in the field face.^15^^–^^17^^,^^58^ Unfortunately, we could not correlate the frequency and quantity of antivenom administration with up-to-date data on antivenom stocks at the facility level because this information was not gathered. Importantly, in the absence of a randomized approach, it is difficult to determine if antivenom slowed, halted, or prevented the occurrence of necrosis or swelling in some patients. Differences in the severity of envenoming between the antivenom and no-antivenom groups could imply indication bias because clinicians may have selected more severely envenomed patients for treatment with antivenom. Finally, the observational study design precludes us from drawing conclusions regarding causality.

## CONCLUSION

This is the first multicenter prospective clinical observational study on snakebites from Eswatini, including snakebite patients from two consecutive snakebite seasons and nine different health facilities with a single antivenom product in use. No measurable differences in the rate of swelling reduction were observed between patients who received antivenom and those who did not. Necrosis developed in several patients despite earlier antivenom therapy. It must be stressed that the shortcomings of an observational study design, the incomplete follow-up, the unknown proportion of different snake species causing the bites, bites caused by species not covered by the antivenom used, and variations in antivenom dosing mandate caution when interpreting these data. The costs of antivenom, its potential serious side effects, and its questionable efficacy in averting local tissue complications are strong arguments for supporting a clinical trial to investigate its efficacy in preventing local tissue complications, preferably stratified by snake species.

## Supplemental Materials

10.4269/ajtmh.24-0671 Supplemental Materials
